# Local striatal volume and motor reserve in drug-naïve Parkinson’s disease

**DOI:** 10.1038/s41531-022-00429-1

**Published:** 2022-12-05

**Authors:** Seong Ho Jeong, Eun-Chong Lee, Seok Jong Chung, Hye Sun Lee, Jin Ho Jung, Young H. Sohn, Joon-Kyung Seong, Phil Hyu Lee

**Affiliations:** 1 Department of Neurology, Yonsei University College of Medicine, Seoul, South Korea; 2 Department of Neurology, Inje University Sanggye Paik Hospital, Seoul, South Korea; 3 School of Biomedical Engineering, Korea University, Seoul, South Korea; 4 Department of Neurology, Yongin Severance Hospital, Yonsei University Health System, Yongin, South Korea; 5 Biostatistics Collaboration Unit, Yonsei University College of Medicine, Seoul, South Korea; 6 Department of Neurology, Inje University Busan Paik Hospital, Seoul, South Korea; 7 Department of Artificial Intelligence, Korea University, Seoul, South Korea; 8 Severance Biomedical Science Institute, Yonsei University College of Medicine, Seoul, South Korea

**Keywords:** Parkinson's disease, Prognostic markers, Basal ganglia

## Abstract

Motor reserve (MR) may explain why individuals with similar pathological changes show marked differences in motor deficits in Parkinson’s disease (PD). In this study, we investigated whether estimated individual MR was linked to local striatal volume (LSV) in PD. We analyzed data obtained from 333 patients with drug naïve PD who underwent dopamine transporter scans and high-resolution 3-tesla T1-weighted structural magnetic resonance images. Using a residual model, we estimated individual MRs on the basis of initial UPDRS-III score and striatal dopamine depletion. We performed a correlation analysis between MR estimates and LSV. Furthermore, we assessed the effect of LSV, which is correlated with MR estimates, on the longitudinal increase in the levodopa-equivalent dose (LED) during the 4-year follow-up period using a linear mixed model. After controlling for intracranial volume, there was a significant positive correlation between LSV and MR estimates in the bilateral caudate, anterior putamen, and ventro-posterior putamen. The linear mixed model showed that the large local volume of anterior and ventro-posterior putamen was associated with the low requirement of LED initially and accelerated LED increment thereafter. The present study demonstrated that LSV is crucial to MR in early-stage PD, suggesting LSV as a neural correlate of MR in PD.

## Introduction

Parkinson’s disease (PD) is a neurodegenerative disorder characterized by a progressive decline in motor performance associated with the loss of dopaminergic neurons in the substantia nigra^1^. The motor symptoms in patients with PD become clinically obvious only after extensive dopaminergic neuronal degeneration and a 70% decrease in striatal dopamine concentration^2^. Thus, the pre-symptomatic stage of PD must involve compensatory mechanisms that allow substantial dopamine depletion to take place without symptomatic manifestations^3–5^. In patients with Alzheimer’s disease (AD), evidence suggests that the compensatory ability to cope with AD pathology exists, which is called “Cognitive reserve”^6^. Similarly, the concept of motor reserve (MR) has been proposed to explain the ability to cope with the pathology of PD, and the presence of MR can explain the individual differences in parkinsonian motor deficits at similar levels of pathological changes^7,8^. Moreover, initial MR is an important factor that modulates longitudinal motor prognosis, implicating therapeutic strategies^8,9^.

The exact mechanism of MR in PD is largely unknown; however, patterns of nigrostriatal dopamine depletion^10,11^, the severity of white matter hyperintensities^12^, and MR network comprised of the basal ganglia, inferior frontal cortex, insula, cerebellum, hippocampus, and amygdala^9^ have been suggested. Previous studies demonstrated that premorbid exercise engagement, education attainment, and side-of-onset were associated with MR in patients with PD^8^. Interestingly, these clinical factors are related to the volume of the subcortical structure, mainly striatum^13–15^ and the pattern of change in striatal volume appears to be associated with motor deficits in spite of inconsistent results^16–18^. In the present study, we hypothesized that the local striatal volume (LSV) would be a neural correlate of MR in patients with early-stage PD. To demonstrate this, we performed a surface-based shape analysis procedure to determine whether a large LSV was closely coupled with high estimated individual MR among individuals with drug-naïve PD. Additionally, we analyzed longitudinal changes in levodopa-equivalent dose (LED) to confirm that the striatal area related to MR can predict longitudinal motor prognosis among patients with PD.

## Results

### Baseline demographic characteristics

The demographic and clinical characteristics of patients with PD enrolled in the present study are shown in Table 1. The mean age of symptom onset was 63.99 ± 9.69 years, and the mean disease duration was 16.87 ± 15.23 months. The mean Unified PD Rating Scale Part III (UPDRS-III) score at the time of diagnosis was 23.10 ± 10.45. More than 75% of PD patients were akinetic-rigid or mixed type. As expected, tremor-dominant PD patients had lower UPDRS-III scores, higher DAT availability, and higher MR estimates than akinetic-rigid or mixed-type PD patients. The striatal volume was comparable between the tremor-dominant PD and akinetic-rigid or mixed PD groups.

**Table 1:** Demographic characteristics.

Variables	Patients with PD (*n* = 333)	TD type (*n* = 76)	AR or mixed type (*n* = 257)	*p*^a^ value
Age at symptom onset, years	63.99 ± 9.69	62.25 ± 9.81	64.50 ± 9.62	0.075
Female subjects, no. (%)	177 (53.1%)	37 (48.7%)	140 (54.5%)	0.449
Disease duration, months	16.87 ± 15.23	18.41 ± 15.81	16.45 ± 15.09	0.326
UPDRS-III score	23.10 ± 10.45	20.29 ± 9.66	23.93 ± 10.55	0.007
Education, years	9.49 ± 5.09	10.58 ± 4.85	9.17 ± 5.13	0.035
Striatal volume, ratio to ICV				
Right caudate	0.21 ± 0.03	0.21 ± 0.03	0.21 ± 0.03	0.383
Right putamen	0.28 ± 0.04	0.29 ± 0.05	0.28 ± 0.04	0.260
Left caudate	0.20 ± 0.03	0.20 ± 0.03	0.20 ± 0.03	0.366
Left putamen	0.28 ± 0.04	0.28 ± 0.05	0.27 ± 0.04	0.119
DAT availability				
Anterior caudate	2.12 ± 0.69	2.25 ± 0.60	2.08 ± 0.71	0.067
Posterior caudate	1.42 ± 0.57	1.52 ± 0.47	1.39 ± 0.59	0.049
Anterior putamen	2.23 ± 0.64	2.41 ± 0.58	2.18 ± 0.65	0.006
Posterior putamen	1.38 ± 0.47	1.49 ± 0.45	1.35 ± 0.47	0.015
Ventral putamen	1.48 ± 0.43	1.58 ± 0.39	1.45 ± 0.43	0.014
Ventral striatum	2.15 ± 0.61	2.30 ± 0.57	2.10 ± 0.61	0.015
MR estimates		0.19 ± 0.57	−0.07 ± 0.61	0.048

*AR* akineto-rigid, *DAT* dopamine transporter, *ICV* intracranial volume, *PD* Parkinson’s disease, *TD* tremor-dominant, *UPDRS-III* Unified Parkinson’s Disease Rating Scale part III.

^a^Comparison between TD type and AR or mixed type.

### Calculation and validation of MR estimate

A general linear model demonstrated that predicted UPDRS-III scores were significantly and positively associated with age (*β* = 0.266, *p* < 0.001) and disease duration (*β* = 0.125, *p* < 0.001) and were negatively associated with the natural logarithm of DAT availability in the posterior putamen (*β* = −6.782, *p* < 0.001) (Table 2). After estimating individual MR using the residual model, we validated MR estimates with education attainment, which is a well-known MR proxy^19^. MR estimates were positively correlated with years of education (correlation coefficient (*r*) = 0.144, *p* = 0.009); participants with high levels of education had high MR.

**Table 2:** General linear model for the prediction of UPDRS-III scores.

Variables	*β*	Standard error	*p* value
Intercept	5.449	4.007	0.174
Age	0.266	0.055	<0.001
Sex	0.176	1.078	0.870
Disease duration	0.125	0.035	<0.001
ln (DAT availability)^a^	−6.782	1.532	<0.001

*DAT* dopamine transporter, *UPDRS-III* Unified Parkinson’s Disease Rating Scale part III.

^a^Because the dopamine transporter availability in the posterior putamen was not normally distributed, its natural logarithm was used in the general linear model.

### LSV and baseline UPDRS-III score

Partial correlation analyses conducted to investigate the correlation between LSV (i.e. right caudate, right putamen, left caudate, and left putamen) and baseline UPDRS-III score after adjusting for age, sex, disease duration, and ICV showed no significant regions. There were still no areas showing a significant correlation between LSV and UPDRS-III score after adding DAT availability in the posterior putamen as a covariate. Similarly, there was no significant correlation between LSV and each UPDRS-III subscore (i.e., tremor, rigidity, bradykinesia, or axial symptoms).

### Striatal volume and MR estimates

Correlation analyses were conducted to investigate the relationship between each striatal volume and MR estimates. There was no association between MR estimates and striatal volume in the right caudate (*r* = −0.019, *p* = 0.736), right putamen (*r* = −0.007, *p* = 0.902), left caudate (*r* = −0.010, *p* = 0.857), and left putamen (*r* = −0.010, *p* = 0.850).

### LSV and MR estimates

**Fig. 1:**
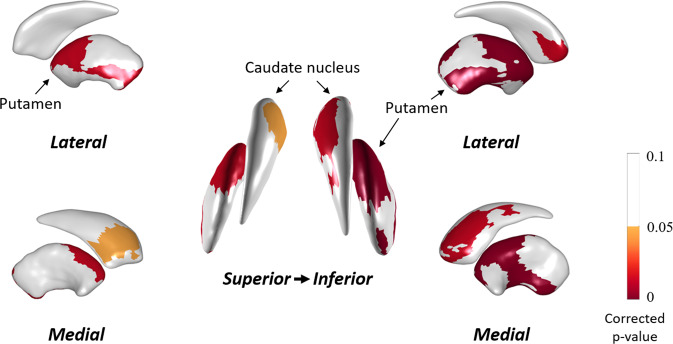
Statistical maps of the striatal local region that correlates with MR estimates with ICV adjustment. Pearson’s correlation analyses showed that bilateral anterior and ventro-posterior putamen and bilateral caudate local volume significantly correlate with MR estimates. The mapped *p* values are the results after multiple comparisons correction using cluster-based statistics. Red represents the degree of significance of correlation coefficients.

Partial correlation analyses were performed to investigate the correlation between LSV and MR estimates while controlling for intracranial volume (ICV). There was a significant positive correlation between MR estimates and LSV in the bilateral caudate and anterior and ventro-posterior putamen (Fig. 1). In addition, to exclude the existence of dopaminergic effects on MR estimates, correlation analysis was performed to uncover the correlation between DAT availability in the anterior putamen and MR estimate. In further analysis, we investigated whether there are between-group (tremor dominant type vs akinetic-rigid or mixed type) differences in the relationship between MR estimates and LSV using interaction analysis. The interaction term was not significant, which indicates that there was no group effect on the relationship between the MR estimate and the LSV.

### LSV and DAT availability

Partial correlation analysis between DAT availability in the bilateral striatal subregions (anterior caudate, posterior caudate, anterior putamen, posterior putamen, ventral putamen, and ventral striatum) and LSV while adjusting for age, sex, disease duration, and ICV showed no significant association between the variables (Supplementary Fig. 1).

### DAT availability and MR estimates

**Fig. 2:**
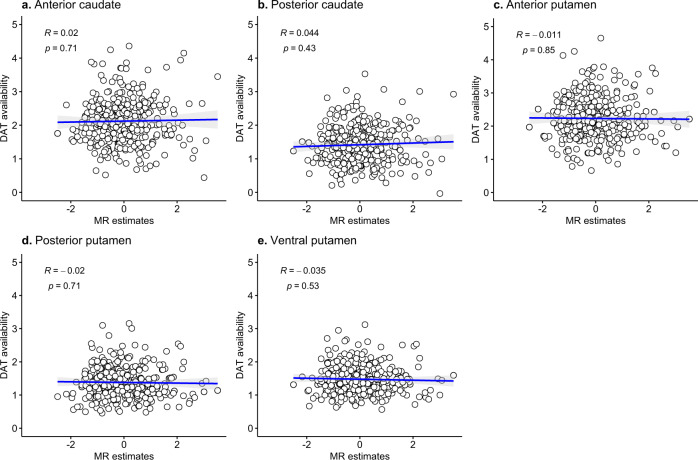
Correlation analysis between DAT availability and MR estimates. **a**–**e** DAT availability in each striatal subregion was not associated with MR estimates.

There was no association between MR estimates and DAT availability in the anterior caudate (*r* = −0.028, *p* = 0.609), posterior caudate (*r* = −0.053, *p* = 0.338), anterior putamen (*r* = 0.002, *p* = 0.966), ventral putamen (*r* = 0.026, *p* = 0.639), and ventral striatum (*r* = −0.00002, *p* > 0.999, Fig. 2).

### LSV and longitudinal motor progression

**Fig. 3:**
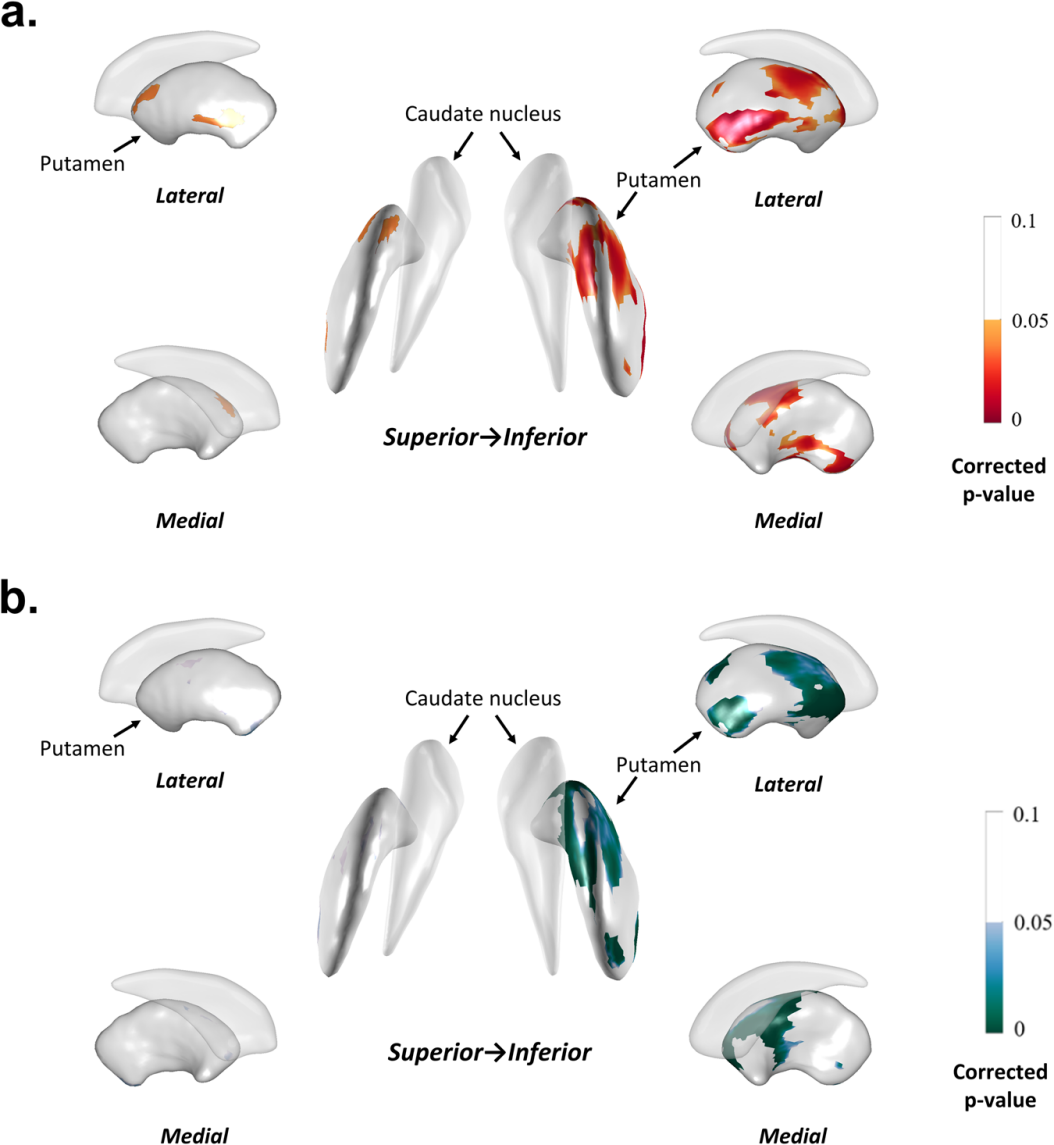
Local striatal volume and longitudinal LED increase. **a** Linear mixed model revealed that right anterior and ventro-posterior putamen local volume showed significant interaction with time for LED increment within 24 months, which means that the larger the local volumes of structures noted above, the slower the rate of LED increment. **b** However, from 24 months to 48 months, the results were opposite and the large local volume of right anterior and ventro-posterior putamen correlates with a rapid increment of LED. The mapped *p* values are the results after multiple comparisons correction using the false discovery rate method.

Subsequently, we analyzed the linear mixed model to examine the relationship between LSV and longitudinal motor progression. The equation of the linear mixed model was given by Eq. (1).

1 $$\begin{array}{l}{{{\mathrm{LED}}}}\sim \beta _0 + \beta _1 \times {\mathrm{time}} + \beta _2 \times {\mathrm{striatal}}\;{\mathrm{LSV}} + \beta _3 \times {\mathrm{time}} \times {\mathrm{striatal}}\;{\mathrm{LSV}} + \\ 1\;{\mathrm{participant}} + {\mathrm{covariates}}\end{array}$$

where covariates include the age of symptom onset, sex, disease duration, and group (tremor dominant type vs akinetic-rigid or mixed type). There was a significant interaction between LSV and time for longitudinal changes in LED after adjusting for age, sex, disease duration, and group (tremor dominant type vs akinetic-rigid or mixed type) The result showed that larger LSV of bilateral anterior and ventro-posterior putamen attenuated the increase of LED over time regardless of age, sex, disease duration, and group for 24 months (Fig. 3a). In contrast, patients with a larger volume of the right anterior and ventro-posterior putamen showed an accelerated increment of LED from 24 months to 48 months (Fig. 3b).

## Discussion

In this study, we investigated the relationship between LSV and MR in patients with drug naïve PD. The major findings were as follows: (1) MR estimates were positively correlated with the LSV of bilateral striatum, especially the head of the caudate, anterior, and ventro-posterior putamen; (2) MR estimates had no significant relationship with dopamine transporter availability in the striatum whose LSV was correlated with MR estimates; and (3) MR-associated striatal structures played a significant role in patterns of longitudinal LED requirement; in patients with larger LSV, the increase of LED requirement was attenuated for initial 24 months and was accelerated thereafter.

Generally, the severity of parkinsonian motor symptoms is well correlated with nigrostriatal dopamine depletion. However, the individual difference in the capacity to cope with PD pathology causes marked variation in parkinsonian motor deficits despite a similar degree of dopaminergic degeneration on DAT images. In this study, we estimated the baseline MR of each patient using a residual model as in our previous studies. We also validated the MR estimate by showing a significant correlation with a previously proposed MR proxy, education attainment. Regarding the compensation against motor deficits, presymptomatic mechanisms in patients with PD were believed to be mediated by dopamine, such as increased synthesis, release, and turnover of dopamine and reduced dopamine reuptake^4,20–22^. However, recent studies suggested that the compensation may occur through extra-dopaminergic pathway in patients with PD^3^.

The present study showed that the UPDRS-III score was not significantly associated with the LSV. The relationship between parkinsonian motor deficits and LSV or morphology is controversial. Three previous studies, which investigated the association between the UPDRS-III score and LSV, failed to show the association^23–25^. In contrast, Nemmi et al. showed that the UPDRS right motor scores were associated with left putamen LSV^18^. Moreover, the morphometric analysis demonstrated a significant relationship between the UPDRS-III score and gray matter reduction in the striatum^16^. Different covariates in correlation analysis and different methods (i.e. shape analysis, volumetry, or gray matter intensity) in each study may lead to discordant results. Also, considering that presynaptic dopaminergic neuronal loss is the major determinant of parkinsonian motor deficits, future studies should consider this variable in the analyses.

In this study, a surface-based shape analysis showed that the LSVs of bilateral caudate, anterior, and ventro-posterior putamen were significantly correlated with MR estimates. It suggests that a larger LSV is associated with an individual’s higher motor compensatory ability in patients with PD. There is a growing body of evidence demonstrating a morphological change in subcortical structures in PD^23,25^. One study found that bilateral putamen volume was significantly reduced in early-stage PD^23^, and autopsy-based studies reported neuronal loss and dendritic degeneration in the striatum, especially the putamen^26,27^. A recent study demonstrated a significant local atrophic change in the dorso-posterior putamen where decoupling between substantia nigra mainly occurs in PD pathology^28^. However, the clinical relevance of the changes in the striatum in PD is unknown so far. In this study, with a large number of patients with early-stage PD, we demonstrated for the first time that the LSVs in the bilateral caudate, anterior, and ventro-posterior putamen may be neuroanatomical correlates of MR in patients with PD. This result is in line with previous studies showing that the anterior subunit of the striatum may play a crucial role in disease progression and motor compensation because the posterior putamen must already have undergone prominent deafferentation from nigrostriatal dopaminergic neurons for the disease to become apparent^29,30^. Additionally, Helmich et al. found an anterior shift of functional connectivity between the sensorimotor cortex and striatum in patients with PD in carriers of asymptomatic LRRK2 G2019S mutation^31,32^. In our study, considering that there was no relationship between DAT availability in the caudate, anterior and ventral putamen and MR estimates, the caudate and putamen as a MR-related structure may perform a role through extra-dopaminergic mechanisms. However, because DAT availability in the posterior putamen is already exhausted when patients with PD visit a clinic for motor complaints, it is plausible that dopaminergic mechanisms play a role in the prediagnostic (i.e., preclinical or prodromal) phase of PD, while extra-dopaminergic mechanisms emerge in the clinical phase of PD.

**Fig. 4:**
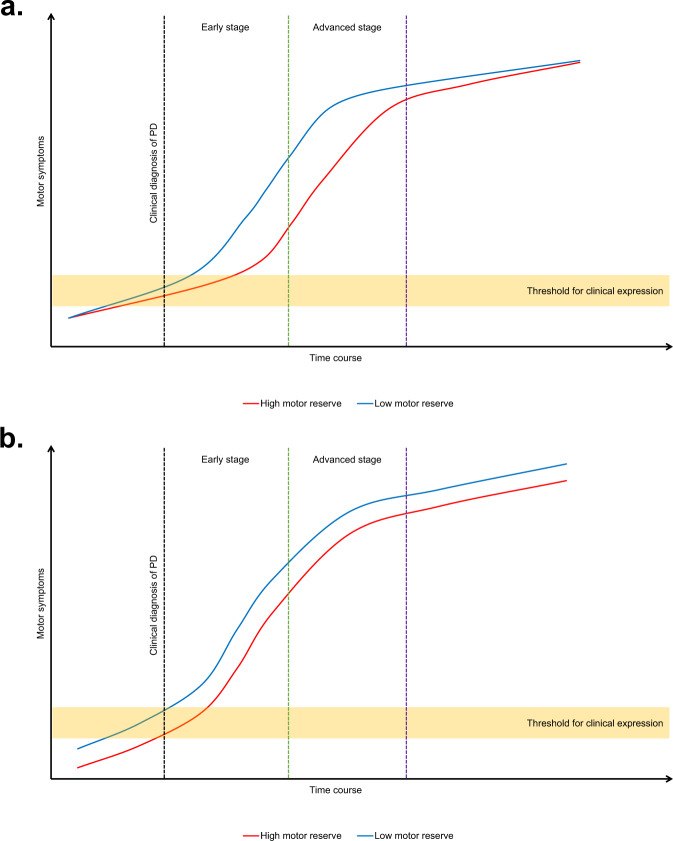
Two possible mechanisms of motor reserve in Parkinson’s disease. **a** An active motor reserve hypothesis shows that the rate of motor symptom progression is different according to the disease stage. **b** In contrast, a passive motor reserve model shows the progression rate of motor symptoms is not affected by the disease stage.

Interestingly, the linear mixed model showed that a larger LSV of the anterior and ventro-posterior putamen was associated with a slower rate of increase in LED requirement for the control of parkinsonian motor symptoms during the first 2 years of dopaminergic medication, however, LED requirement was significantly accelerated thereafter. Our results suggest that PD patients with high MR eventually seem to have a rapid deterioration of motor symptoms than those with low MR and anterior and ventro-posterior putamen plays a role in acting as a neural correlate of MR. This finding raises the possibility that MR observed in this study may be an active reserve rather than a passive reserve (Fig. 4). There are at least two models that explain reserve in cognition, namely brain reserve and cognitive reserve^33^. In the brain reserve model, the reserve is conceived as a passive entity, and the progression rate does not differ according to the level of brain reserve^34^. In contrast, the cognitive reserve model is regarded as an active entity and affected by educational and physical activity^33^. In this model, the presence of a high cognitive reserve attenuates cognitive decline in pre-dementia stages, then accelerated clinical progression after the onset of dementia^35^. Although these two models are not mutually exclusive, several studies have supported a passive reserve hypothesis in patients with PD^36,37^. The result of Park-in-Shape trial showed that aerobic exercise increased functional connectivity between the sensorimotor cortex and the anterior putamen rather than the posterior putamen^38^, which supports the result of the present study that the local volume of anterior and ventro-posterior putamen is crucial for active reserve in patients with PD. The present study was the first to show a possible active MR mechanism involving local striatal volume. Further large-scale longitudinal studies are necessary to draw a more solid conclusion.

Previously, we demonstrated that functional connectivity within basal ganglia, inferior frontal cortex, insula, and cerebellar vermis plays a crucial role in MR in patients with PD^9^. However, functional MRI is not always available in routine clinical practice due to its time-consuming protocol. In this study, we enrolled larger participants than in the previous study using high-resolution T1 MR images, which are more easily acquirable than functional MRI. Considering that structural alternation may be associated with the change of functional connectivity^39,40^, our results suggest that LSV change in the striatum may reflect the functional change of MR network and may also be useful for an imaging biomarker of MR in patients with PD.

There are several limitations to this study. First, we assessed individual MR estimates using DAT availability in the posterior putamen. Downregulation of DAT can occur as a compensatory mechanism to maintain dopamine levels in the synaptic clefts in patients with early-stage PD^41^, and thus, in vivo DAT availability may not reflect nigrostriatal degeneration accurately. Second, nonlinear partial-volume effects may underestimate the density of radioactivity in small lesions^42^, especially when using a PET scanner with a poor spatial resolution (5 to 6 mm full-width-half-max [FWHM])^43^. However, this study used high-resolution PET images with 2.3 mm FWHM, which may minimize this issue. Third, even though a consensus for clinically meaningful endpoints has not been established, LED and its longitudinal changes for the control of parkinsonian motor symptoms may indirectly reflect the severity of PD and disease progression markers^44^. However, because global disability in patients with PD is complexly influenced by motor and non-motor symptoms^45,46^, an increase in LED requirement may not accurately reflect the progression of PD. Fourth, the residual model has a clear limitation in that residuals from the general linear model are inevitably correlated with their dependent variables^35^. Therefore, we confirmed that the correlation between MR estimates and striatal structures is independent of UPDRS-III score by showing no relationship between UPDRS-III and LSV. Additionally, residual models may contain information about prodromal disease duration and individual progression rate in addition to MR. However, there is still no way to measure prodromal disease duration or progression rate at baseline. If future studies develop a method measuring these variables, these should be considered to estimate MR in patients with PD. Finally, attrition bias may occur between 2 to 4 years in the longitudinal analysis; we attempted to minimize this bias using a linear mixed model to fit longitudinal data in the presence of nonrandom dropout.

In summary, the present study demonstrated that LSV is crucial in MR in patients with early-stage PD, which indicates a capacity to cope with neurodegenerative processes. Our findings suggest that LSV can be a neural correlate associated with an individual’s motor compensatory ability.

## Methods

### Participants

This retrospective cohort study included 339 patients with drug naïve PD who visited the movement disorders outpatient clinic at Yonsei University Severance Hospital between April 2009 and September 2015. Among these patients, six patients were excluded because of errors in the image preprocessing steps. Thus, data obtained from 333 patients were used in this analysis. PD was diagnosed according to the clinical diagnostic criteria of the U.K. PD Society Brain Bank. All patients underwent dopamine transporter (DAT) Imaging using [^18^F] *N*-(3-fluoropropyl)-2β-carbonethoxy-3β-(4-iodophenyl) nortropane positron emission tomography (^18^F-FP-CIT PET), which revealed a decrease in DAT availability in the posterior putamen on all the subjects^47^. In addition, they underwent magnetic resonance imaging (MRI), including high-resolution T1-weighted MRI at baseline. Parkinsonian motor symptoms were evaluated using the UPDRS-III at the initial visit. Subscores for tremor (UPDRS, items 20 and 21), rigidity (item 22), bradykinesia (items 18, 19, 23–26, and 31), and axial symptoms (items 27–30) were also calculated^48^. Patients with PD were accordingly classified into three clinical subtypes: tremor dominant (TD), akinetic-rigid (AR), and mixed^49^. This study was approved by the Yonsei University Severance Hospital institutional review board, and the requirement for written informed consent was waived because of the retrospective nature of the study.

### Quantitative analyses of ^18^F-FP-CIT PET

The ^18^F-FP-CIT PET images were acquired using a GE PET-CT DSTe scanner (GE Discovery STE; GE Healthcare; Milwaukee, WI, USA), which obtains images with a three-dimensional resolution of 2.3-mm full width at half maximum. After the subjects fasted for at least 6 h, they were intravenously injected with 5 mCi (185 MBq) of ^18^F-FP-CIT. 90 min after the injection, PET images were acquired for 20 min in the three-dimensional mode at 12 kVp and 380 mA. Image processing was performed using SPM8 (Wellcome Department of Imaging Neuroscience, Institute of Neurology, UCL, London, UK) with Matlab 2013a for Windows (Math Works, Natick, MA, USA). Quantitative analyses were based on volumes of interest (VOIs), which were defined on the basis of a template in standard space. All reconstructed PET images were spatially normalized to the Montreal Neurology Institute (MNI) template space using a standard ^18^F-FP-CIT PET template which was generated in-house from ^18^F-FP-CIT PET and T1-weighted MRI scans of 13 normal controls (four men and nine women, mean age 55.2 ± 9.2 years) as described previously to remove intersubject anatomic variability^50^. All healthy controls had no previous history of psychiatric or neurologic illness and showed normal cognition on all neuropsychological items. First, we co-registered individual ^18^F-FP-CIT PET images onto corresponding T1-weighted MRI images. Second, each individual T1-weighted MRI image was spatially normalized onto the MNI T1 template. Third, the deformation fields, which are used to map the individual MRI images to the MNI T1 template, were applied to the corresponding ^18^F-FP-CIT PET images. Finally, the ^18^F-FP-CIT PET template was defined by averaging the spatially normalized ^18^F-FP-CIT PET images.

Twelve VOIs of bilateral striatal subregions and one occipital VOI were drawn on a co-registered spatially normalized single T1-weighted magnetic resonance and ^18^F-FP-CIT PET template image on MRIcro version 1.37 (Chris Rorden, Columbia, SC, USA)^47^. To describe briefly, the striatum was divided along the anterior-posterior commissure line on the transverse plane into dorsal and ventral portions. The ventral portion comprises two subregions: the ventral putamen and the ventral striatum. Subsequently, the dorsal portion was divided along the coronal anterior commissure plane into the following anterior and posterior subregions: the anterior caudate, posterior caudate, anterior putamen, and posterior putamen. The positions of the automatically defined template VOIs were adjusted manually by our in-house VOI editing software called ANIQUE (AMC NM Image Quantification Toolkit of Excellence) to ensure registration accuracy^51^. Our manual adjustment of VOI is a step that may minimize possible mis-registration occasionally occurring in the automated VOI analysis. DAT availability was calculated using the non-displaceable binding potential, which was defined as follows: (mean standardized uptake value of the striatal subregions VOI–mean standardized uptake value of the occipital VOI)/(mean standardized uptake of the occipital VOI)^52^.

### MRI acquisition

All scans were acquired with a Philips 3.0 T scanner (Philips Intera; Philips Medical System, Best, The Netherlands) with a SENSE head coil (SENSE factor = 2). A high-resolution, T1-weighted MRI volume data set was obtained from all subjects with a 3-dimensional T1-TFE sequence configured with the following acquisition parameters: axial acquisition with a 224 × 256 matrix; 256 × 256 reconstructed matrix with 182 slices; 220-mm field of view; 0.98 × 0.98 × 1.2 mm^3^ voxels; 4.6 ms echo time; 9.6 ms repetition time; 8° flip angle; and 0 mm slice gap.

### Assessment of striatal volume and LSV

The volume of the caudate and putamen on each side was extracted. These values were divided by ICV to normalize for individual brain size. The assessment of LSV was performed by measuring the local shape on the striatal surface meshes^53^. The whole process consists of the following four steps: volume parcellation, surface extraction, registration, and local atrophy computation. In the first step, subcortical structures were parceled from T1 images of each subject with the FreeSurfer software package (version 6.0.0; Athinoula A. Martinos Center at the Massachusetts General Hospital, Harvard Medical School; http://www.surfer.nmr.mgh.harvard.edu/) with the Desikan–Killiany atlas in MNI 152 space. The parceled volumes were transformed into native anatomical space for surface extraction. In the second step, a surface mesh was extracted for each subject by deforming the template surface atlas models^54^, using the Laplacian-based surface deformation method^55^. In the third step, the surface registration method developed by Cho et al.^56^ was applied to establish the vertex correspondence of striatal surface meshes across the sample. In the final step, the local shape of each vertex was measured by employing the surface-based method proposed by Shapira et al.^57^. In detail, rays forming an angle of 60 ° or less with the inner normal direction were shot for each vertex on the surface mesh. Then, when they hit the opposite mesh surface, we measured the lengths of the rays and combined them as a weighted sum. Approximately 600 ray shootings were performed for each vertex, and their weights were calculated using the angle formed with the inner normal vector. This definition implies that the smaller this measure, the greater the degree of local contraction or local shrinkage at each vertex. Therefore, this measure was designated as the local volume in this study. Through a series of processes, local volume values on 2562 vertices for each striatum were calculated. Volumes of each of the four striatal structures and intra-cranial volume were obtained on the basis of anatomical image segmentation using the FreeSurfer software package.

### Longitudinal changes in dopaminergic medication doses

**Fig. 5:**
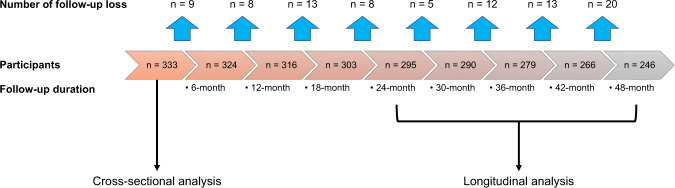
Attrition rate in the cohort. In this study, 333 patients were enrolled in the cross-sectional analysis, and 295 patients who were followed-up more than 2 years were enrolled in the longitudinal analysis.

Information on attrition in the cohort over time is shown in Fig. 5. A subset of participants (*n* = 295) visited the outpatient clinic for at least 2 years, and these patients, with the exception of one, underwent measurement of the LED values every 6 months up to 4 years (*n* = 294). The doses of their dopaminergic medications were adjusted to control motor symptoms effectively by L.P.H. and S.Y.H. according to the patients’ responses. If the patients had intolerable dyskinesia or adverse effects from pharmacotherapy, no titration of the medications for PD was performed, and the assessment of LED changes was continued only up to that point. We calculated the LED of dopaminergic medication based on a previously reported method^58^. We then performed a linear mixed model to investigate whether the LSV which was significantly correlated with MR estimates modified the longitudinal change in LED over time.

### Estimation of MR

MR estimation was performed as described previously^9^. In detail, we used the general linear model to predict the UPDRS-III score by using age, sex, disease duration, and the natural logarithm of DAT availability in the posterior putamen (Table 2). Subsequently, the residuals (i.e., differences between the actual value and the predicted value of UPDRS-III score) in the general linear model were calculated and standardized^8,9^. A greater standardized residual indicated that the subject had a greater UPDRS-III score than the predicted score (i.e., lower MR). We defined a negative value of the standardized residuals as the “MR estimate” of each patient and higher values indicate higher MR, which means that the actual UPDRS-III score of the subject is lower than the UPDRS-III predicted score.

### Statistical analysis

We calculated Pearson’s correlation coefficient to identify the relationship between LSV and MR, and between LSV and UPDRS-III score. All analyses were performed at the vertex level of the subcortical surface for both negative and positive correlations, and the results were reported after multiple corrections. To investigate the association of LSV and UPDRS-III score in patients with PD, Pearson’s correlation analysis was performed by adjusting with age, sex, disease duration, and ICV. To evaluate the correlation of LSV and MR estimates, Pearson’s correlation coefficient was estimated adjusting only for ICV, because MR estimates are residuals obtained from the general linear model adjusted for age, sex, disease duration, and the natural logarithm of DAT availability in the posterior putamen. To exclude a circular relationship between DAT availability, LSV, and MR estimates, we further performed a partial correlation analysis between DAT availability in each striatal subregion (anterior caudate, posterior caudate, anterior putamen, ventral putamen, and ventral striatum) and LSV while adjusting for age, sex, disease duration, and ICV. Because MR estimates are the result after adjusting for DAT availability in the posterior putamen, correlation analysis between MR estimates and DAT availability in the posterior putamen was not performed. In addition, the presynaptic dopaminergic effect on MR estimates was assessed using correlation analysis between DAT availability in each striatal subregion and MR estimate. The region that has a stronger negative correlation of −0.3 or a stronger positive correlation of 0.3 was selected to indicate a moderate association between the two variables^59^. We used cluster-based statistics with 5,000 iterations for multiple corrections independently for each structure of the bilateral striatum. After obtaining a partial correlation coefficient for 5,000 permutations, the *p* value for the sum of the correlation coefficients in each cluster with a stronger correlation than the threshold was calculated. In other words, we obtained the maximum value of the sum of the correlation coefficients for each permutation. The level of significance was then calculated for the clusters in the original data.

In addition, a linear mixed model was built to investigate the within-striatal region that has a relationship with the longitudinal LED increment for each individual. It was also performed at the vertex level of the subcortical surface. As for the LSV used in this study, only the LSV showing a significant correlation with MR (mainly bilateral anterior caudate and anterior and ventro-posterior putamina) was selected. The equation of the linear mixed model was presented in Eq. (1), as aforementioned.

In this model, subjects were added as random effects, and age, sex, and disease duration were added as fixed effect terms. The effect of LSV on the longitudinal LED increment was tested through the interaction term between time and LSV. To test for the interaction term, we used a false discovery rate (FDR) multiple corrections for each striatal structure, because the correction process was only carried out in a specific area of the structure. To separately analyze the short-term and long-term effects of the deformity of the LSV over time on the longitudinal LED increment, we investigated the interaction effects from the date of the diagnosis to 24 months and from 24 to 48 months, respectively. For noise removal, only the significant region with a size larger than 200 vertices were reported in the Results section.

### Reporting summary

Further information on research design is available in the Nature Research Reporting Summary linked to this article.

## Supplementary information

Supplementary Materials

Reporting Summary

## Data Availability

The de-identified data that support the findings of this study are available from the corresponding authors (J.-K.S. and P.H.L.) upon request. The data are not publicly available due to privacy or ethical restriction.
