# Supplementary material for: Local striatal volume and motor reserve in drug-naïve Parkinson’s disease
Source: NPJ Parkinsons Dis. 2022 Dec 5;8:168. doi: 10.1038/s41531-022-00429-1 (PMC9722895; doi:10.1038/s41531-022-00429-1)
Supplement: Supplementary file 1 — Supplementary Materials [file 41531_2022_429_MOESM1_ESM.pdf]

Supplementary Figure 1. Association between LSV and DAT availability in each striatal subregion

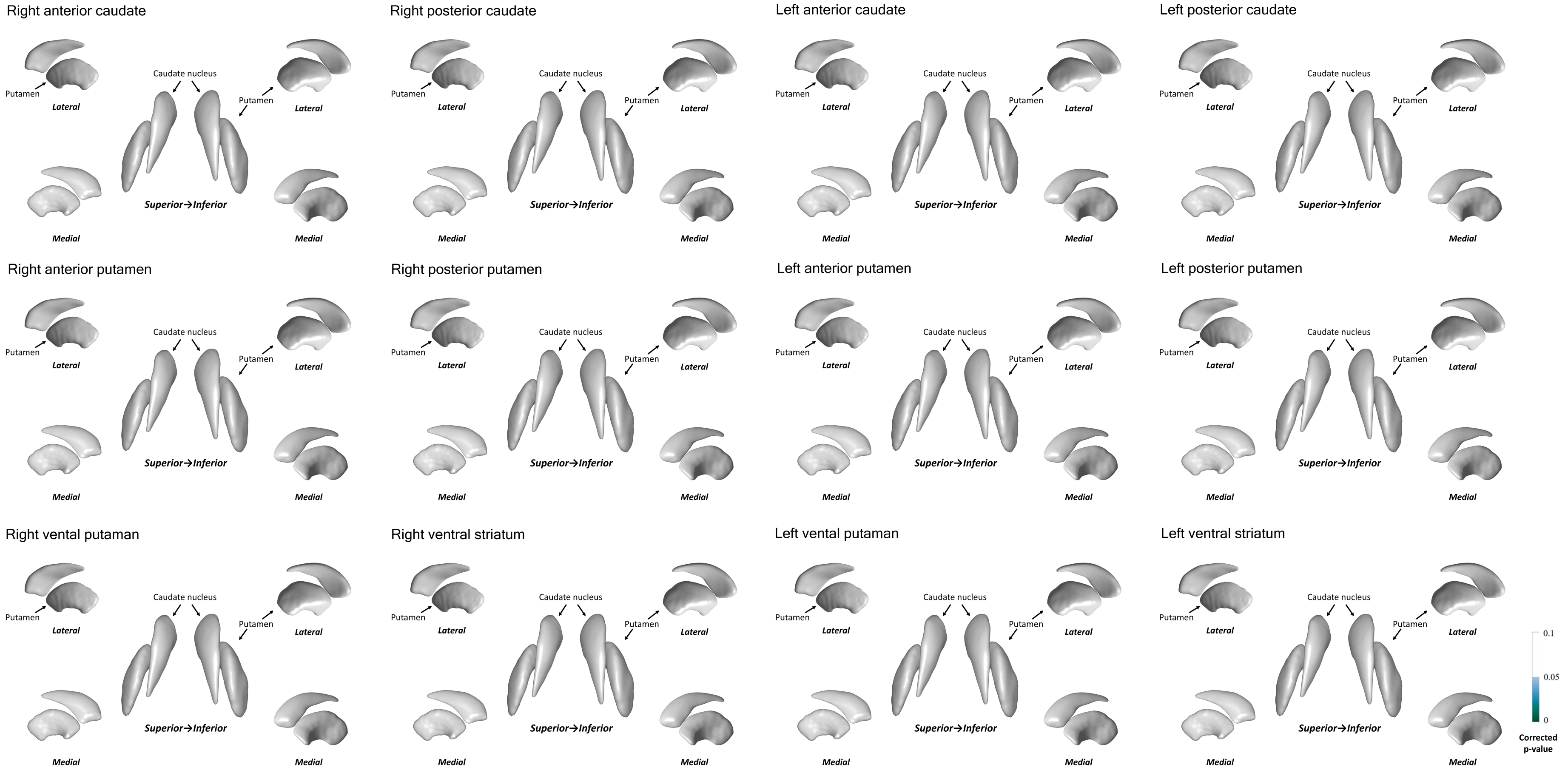

There were no areas of significant correlation between local striatal volume and DAT availability in each striatal subregion.

DAT = dopamine transporter availability
