# Supplementary material for: Local striatal volume and motor reserve in drug-naïve Parkinson’s disease
Source: NPJ Parkinsons Dis. 2022 Dec 5;8:168. doi: 10.1038/s41531-022-00429-1 (PMC9722895; doi:10.1038/s41531-022-00429-1)
Supplement: Supplementary file 2 — Reporting Summary [file 41531_2022_429_MOESM2_ESM.pdf]

nature portfolio

Corresponding author(s): Phil Hyu Lee

Last updated by author(s): Dec 28, 2021

Reporting Summary

Nature Portfolio wishes to improve the reproducibility of the work that we publish. This form provides structure for consistency and transparency in reporting. For further information on Nature Portfolio policies, see our Editorial Policies and the Editorial Policy Checklist.

Statistics

For all statistical analyses, confirm that the following items are present in the figure legend, table legend, main text, or Methods section.

n/a	Confirmed	
☐	☒	The exact sample size (n) for each experimental group/condition, given as a discrete number and unit of measurement
☐	☒	A statement on whether measurements were taken from distinct samples or whether the same sample was measured repeatedly
☐	☒	The statistical test(s) used AND whether they are one- or two-sided<br>*Only common tests should be described solely by name; describe more complex techniques in the Methods section.*
☐	☒	A description of all covariates tested
☐	☒	A description of any assumptions or corrections, such as tests of normality and adjustment for multiple comparisons
☐	☒	A full description of the statistical parameters including central tendency (e.g. means) or other basic estimates (e.g. regression coefficient) AND variation (e.g. standard deviation) or associated estimates of uncertainty (e.g. confidence intervals)
☒	☐	For null hypothesis testing, the test statistic (e.g. F, t, r) with confidence intervals, effect sizes, degrees of freedom and P value noted<br>*Give P values as exact values whenever suitable.*
☒	☐	For Bayesian analysis, information on the choice of priors and Markov chain Monte Carlo settings
☒	☐	For hierarchical and complex designs, identification of the appropriate level for tests and full reporting of outcomes
☐	☒	Estimates of effect sizes (e.g. Cohen's d, Pearson's r), indicating how they were calculated

Our web collection on statistics for biologists contains articles on many of the points above.

Software and code

Policy information about availability of computer code

Data collection	Clinical data were collected based on electrical medical records in Severance Hospital. Image data were converted to DICOM files using Centricity software.
Data analysis	R, MATLAB

For manuscripts utilizing custom algorithms or software that are central to the research but not yet described in published literature, software must be made available to editors and reviewers. We strongly encourage code deposition in a community repository (e.g. GitHub). See the Nature Portfolio guidelines for submitting code & software for further information.

Data

Policy information about availability of data

All manuscripts must include a data availability statement. This statement should provide the following information, where applicable:

- Accession codes, unique identifiers, or web links for publicly available datasets
- A description of any restrictions on data availability
- For clinical datasets or third party data, please ensure that the statement adheres to our policy

The de-identified data that support the findings of this study are available from the authors upon reasonable request.

Field-specific reporting

Please select the one below that is the best fit for your research. If you are not sure, read the appropriate sections before making your selection.

☒ Life sciences ☐ Behavioural & social sciences ☐ Ecological, evolutionary & environmental sciences

For a reference copy of the document with all sections, see nature.com/documents/nr-reporting-summary-flat.pdf

Life sciences study design

All studies must disclose on these points even when the disclosure is negative.

Sample size	*Describe how sample size was determined, detailing any statistical methods used to predetermine sample size OR if no sample-size calculation was performed, describe how sample sizes were chosen and provide a rationale for why these sample sizes are sufficient.*
Data exclusions	*Describe any data exclusions. If no data were excluded from the analyses, state so OR if data were excluded, describe the exclusions and the rationale behind them, indicating whether exclusion criteria were pre-established.*
Replication	*Describe the measures taken to verify the reproducibility of the experimental findings. If all attempts at replication were successful, confirm this OR if there are any findings that were not replicated or cannot be reproduced, note this and describe why.*
Randomization	*Describe how samples/organisms/participants were allocated into experimental groups. If allocation was not random, describe how covariates were controlled OR if this is not relevant to your study, explain why.*
Blinding	*Describe whether the investigators were blinded to group allocation during data collection and/or analysis. If blinding was not possible, describe why OR explain why blinding was not relevant to your study.*

Reporting for specific materials, systems and methods

We require information from authors about some types of materials, experimental systems and methods used in many studies. Here, indicate whether each material, system or method listed is relevant to your study. If you are not sure if a list item applies to your research, read the appropriate section before selecting a response.

Materials & experimental systems

n/a	Involved in the study	
☒	☐	Antibodies
☒	☐	Eukaryotic cell lines
☒	☐	Palaeontology and archaeology
☒	☐	Animals and other organisms
☐	☒	Human research participants
☒	☐	Clinical data
☒	☐	Dual use research of concern

Methods

n/a	Involved in the study	
☒	☐	ChIP-seq
☒	☐	Flow cytometry
☐	☒	MRI-based neuroimaging

Human research participants

Policy information about studies involving human research participants

Population characteristics	The mean age of symptom onset was 63.99 ± 9.69 years, and the mean disease duration was 16.87 ± 15.23 months. 53.1% were women. The mean UPDRS-III score at the time of diagnosis was 23.10 ± 10.45. More than 75% of PD patients were akinetic-rigid or mixed type.
Recruitment	This retrospective cohort study included 339 patients with drug naïve PD who visited the movement disorders outpatient clinic at Yonsei University Severance Hospital between April 2009 and September 2015. Among these patients, six patients were excluded because of errors in the image preprocessing steps. Thus, data obtained from 333 patients were used in this analysis.
Ethics oversight	This study was approved by the Yonsei University Severance Hospital institutional review board, and the requirement for informed consent was waived because of the retrospective nature of the study.

Note that full information on the approval of the study protocol must also be provided in the manuscript.

Magnetic resonance imaging

Experimental design

Design type	N/A
Design specifications	N/A
Behavioral performance measures	N/A

Acquisition

Imaging type(s)	Structural
Field strength	3 Tesla
Sequence & imaging parameters	, T1-weighted MRI volume data set was obtained from all subjects with a 3-dimensional T1-TFE sequence configured with the following acquisition parameters: axial acquisition with a 224 × 256 matrix; 256 × 256 reconstructed matrix with 182 slices; 220-mm field of view; 0.98 × 0.98 × 1.2mm3 voxels; 4.6 ms echo time; 9.6 ms repetition time; 8° flip angle; and 0mm slice gap.
Area of acquisition	Subcortical structure

Diffusion MRI ☐ Used ☒ Not used

Preprocessing

Preprocessing software	FreeSurfer software package
Normalization	N/A
Normalization template	N/A
Noise and artifact removal	For noise removal, only the significant region with size larger than 200 vertices were reported in results.
Volume censoring	N/A

Statistical modeling & inference

Model type and settings	Partial correlation analysis, linear mixed model
Effect(s) tested	Correlation coefficient, regression coefficient

Specify type of analysis: ☐ Whole brain ☐ ROI-based ☒ Both

Anatomical location(s): Subcortical structure using Laplacian-based surface deformation method

Statistic type for inference (See Eklund et al. 2016)	Cluster-wise
Correction	FDR or CBS method

Models & analysis

n/a	Involved in the study
☒	☐ Functional and/or effective connectivity
☒	☐ Graph analysis
☐	☒ Multivariate modeling or predictive analysis

Multivariate modeling and predictive analysis: LED~ β_0 + β_1 × time+ β_2 × striatal LSV + β_0 × time×striatal LSV + 1 participant + covariates
